# A hybrid forecasting model using LSTM and Prophet for energy consumption with decomposition of time series data

**DOI:** 10.7717/peerj-cs.1001

**Published:** 2022-06-10

**Authors:** Serdar Arslan

**Affiliations:** Computer Engineering Department, Cankaya University, Ankara, Turkey

**Keywords:** Time series forecasting, LSTM, Prophet, Hybrid model, Seasonality

## Abstract

For decades, time series forecasting had many applications in various industries such as weather, financial, healthcare, business, retail, and energy consumption forecasting. An accurate prediction in these applications is a very important and also difficult task because of high sampling rates leading to monthly, daily, or even hourly data. This high-frequency property of time series data results in complexity and seasonality. Moreover, the time series data can have irregular fluctuations caused by various factors. Thus, using a single model does not result in good accuracy results. In this study, we propose an efficient forecasting framework by hybridizing the recurrent neural network model with Facebook’s Prophet to improve the forecasting performance. Seasonal-trend decomposition based on the Loess (STL) algorithm is applied to the original time series and these decomposed components are used to train our recurrent neural network for reducing the impact of these irregular patterns on final predictions. Moreover, to preserve seasonality, the original time series data is modeled with Prophet, and the output of both sub-models are merged as final prediction values. In experiments, we compared our model with state-of-art methods for real-world energy consumption data of seven countries and the proposed hybrid method demonstrates competitive results to these state-of-art methods.

## Introduction

Time series forecasting is a popular research area and has various numbers of application fields such as energy, business, economy, health, and environment. Time series forecasting is a process using a dependent variable’s past values to predict its future values. In detail, time series forecasting models try to understand the classical patterns found in these series. These patterns may include seasonality, trend, and noise. The energy field is one of the most studied areas for time series forecasting. As the population of the world grows, energy consumption and demand are always increasing. Moreover, the supply must meet the demand for energy since it cannot be stored. Therefore, the prediction of this consumption and demand has been regarded as a crucial and complex task (Deb et al., 2017).

There are many studies on forecasting energy consumption using various statistical methods or neural networks. Most of these studies are related to specific countries (Mir et al., 2020; Sen, Tunç & Günay, 2021). El-Hendawi & Wang (2020) used full wavelet packet transform and artificial neural networks for short-term electrical load forecasting for the Ontario region of Canada. Similarly, Kaytez (2020) used the least-square vector machine and other statistical methods for annual net electricity consumption in Turkey. For the annual electricity demand prediction of Turkey, the particle swarm optimization algorithm is used in Gulcu & Kodaz (2017). In Di Leo et al. (2020), linear and nonlinear regression methods are used for energy demand forecasting in Italy. Another regression-based model is used in Angelopoulos, Siskos & Psarras (2019) for forecasting annual electricity demand in Greece. Statistical and regression-based methods are combined in Al-Musaylh et al. (2018) for short-term electricity demand forecasting of Australia. In the study proposed in Liu, Moreno & García (2016), annual primary energy consumption is predicted using a combined fore-casting model.

At present, many single statistical methods are also used to forecast energy demand and consumption (De Felice, Alessandri & Catalano, 2015; Jiang, Yang & Li, 2018; Prajapati et al., 2019; Yuan, Liu & Fang, 2016). The auto-regressive integrated moving average (ARIMA) technique which is one of them (Box et al., 1976), however, does not support time series with a seasonal component. Thus, an extension of ARIMA called seasonal ARIMA (SARIMA) has been proposed. In Hyndman & Khandakar (2008), seasonal ARIMA is used with Holt-Winters model. Regression analysis methods are also widely used in other domains (Hong et al., 2020).

Prophet is an open-source time-series forecasting methodology developed by Facebook. Prophet can be seen as a relatively new approach and has become popular among time-series forecasting methodologies because it has ease of usability and power. Moreover, Prophet has been used in several areas already and results show that it outperforms classical statistical and machine learning approaches, especially in the environment field (Vartholomaios et al., 2021; Zhoul, Chenl & Ni, 2020) because of the characteristics of data in these domains. Prophet is designed to handle forecasting on data which has a multiseasonality feature. Thus, Prophet is a good alternative for forecasting seasonal time series data.

Neural networks are also used widely for energy forecasting and these studies can be found in literature surveys (Angelopoulos, Siskos & Psarras, 2019; Hong et al., 2020; Liu et al., 2020; Verwiebe et al., 2021; Wang et al., 2019). Artificial neural networks are used for quarterly energy demand forecasting of Australia, France, the USA (Bannor & Acheampong, 2020), and Greece (Ekonomou, 2010); grey neural networks are modeled for Spain (Liu et al., 2020). The authors in Nam, Hwangbo & Yoo (2020) used recurrent neural net-works (LSTM and GRU) in order to predict electricity demand and generation of South Korea.

Many studies show that seasonal decomposition or adjustment entails better performance results for energy forecasting (Bandara, Bergmeir & Hewamalage, 2021; Heckman, Pinto & Savelyev, 1999; Zhang & Qi, 2005). With seasonal decomposition, the complexity of the original time series is minimized and this reduces the training and learning time of the neural network model (Yan, 2012). Thus, by removing seasonality, neural networks can learn better the other patterns in time series and some studies show that this improves the prediction accuracy (Andrawis, Atiya & El-Shishiny, 2011; Ben Taieb et al., 2012). The study proposed in Zhang & Li (2021) applies the decomposition method for China’s monthly energy consumption data set and SARIMA, SVR, ANN, and LSTM are used to forecast trends, season, and residual components, respectively. They also applied some modifications of residual component for performance improvement. After applying a modification of the residual component, they combined each model output for the final forecast. However, they deal with time series data by dividing them into components and using each component as input for separate models. Another decomposition-based model is presented in Bedi & Toshniwal (2018). They integrated empirical mode decomposition with deep learning methods for forecasting the energy electricity demand of India. They decomposed the original time series into a set of intrinsic mode functions (IMFs) along with a residue. Each IMFs used as input for the regression model and outputs are combined into the final output. Thus, they divided the original time series into several components and these components are used as input for models and treated as independent of each other. Divina et al. (2019) used SVR with differential EMDs for electric load forecasting in Spain.

Both statistical and neural network based approaches have some drawbacks. The statistical approaches have some limitations because of not effectively handling non-linear part of time series while neural network models suffer from overfitting and also have parameter selection/tuning problem. In order to solve these problems, the hybrid models are used widely for forecasting using the time series. Moreover, the prediction accuracy of a single model is limited and dependent on the data set. Hence, the hybridization of models can result improvement in prediction accuracy. In Soy Temür, Akgün & Temür (2019), the authors try to forecast house sales using ARIMA and neural network approach together. For the linear component of the time series they adapted ARIMA model and for the non-linear component, the LSTM model is used. In a similar way, the study proposed in Dave et al. (2021) uses ARIMA and LSTM for export data of Indonesia. A hybrid model using Prophet and LSTM is proposed in Zhoul, Chenl & Ni (2020) for air quality index. In Rodríguez-Rodríguez et al. (2018), an infrastructure-Artificial Intelligence (AI) model with Prophet is proposed to study of a variety of human energy use. In Ji et al. (2019), hybrid forecasting model using ARIMA and deep neural network is proposed for prediction of carbon prices. They used CNN and LSTM for deep neural network model and showed that hybrid model improves the prediction accuracy. In Kamal et al. (2020), the authors proposed three RNN models, Deep RNN, LSTM and GRU, in order to predict the Baltic dry index. These studies show that the results of ensemble models outperform all of the single models but all of them used time series data without extracting the seasonal patterns.

The energy demand/consumption data can have irregular fluctuation caused by various factors. The decomposition of these data can help us reduce the influence of these factors and also can improve the accuracy of demand/consumption prediction by reflecting real world scenarios. Moreover, after decomposition, each component can be modelled independently, so that the model complexity is less than forecasting the original time series as a whole. While statistical methods use different approaches to deal with all components of time series, including seasonality, there is no general approach or methodology to handle seasonality in neural network models. Because these models are nonlinear they should cope with seasonality directly without transforming the time series. However, for artificial neural networks, removal of seasonal properties are recommended to improve forecasting accuracy (Martínez et al., 2018). In this study, we have developed a hybrid forecasting model to eliminate the influence of these factors by using decomposition method. Our model consists of two sub models; Prophet and stacked bidirectional LSTM. In order to preserve seasonality, our study has used Prophet model for original data. On the other hand, bidirectional stacked LSTM is developed for decomposed time series data to reduce the impact of irregular pat-terns. Final predictions are evaluated by combining outcomes of both models. Another contribution of this study is that hybridization of LSTM with Prophet eliminates the shortcomings of each model. The model is tested by using real world energy consumption data for seven (7) different countries and results show that the proposed model outperforms statistical approaches and also, neural network models for prediction. Moreover, the proposed model is compared with state-of-art-techniques and demonstrated satisfactory forecasting results.

This article is organized as follows. In “Forecasting Methods”, forecasting methods are introduced. The proposed hybrid model is discussed in “Proposed Hybrid Model”. “Results” presents experiment results and discussion in which the performance of our model is evaluated. Finally, “Conclusion” draws some conclusions.

## Forecasting Methods

### Prophet

Prophet is an open-source time-series forecasting library developed by Facebook. It uses several distinct methods for time series forecasting. It also sup-ports seasonality and holiday week day split. Prophet consists of three main components. The first component is called trend, and is used to describe the trend in the time series data. The second component is seasonality and the third component is holidays. These three components can be described using the following equation:

${Y_t} = {g_t} + {s_t} + {h_t} + {{ \epsilon }_t}$

where, ${g_t}$ represents trend, ${s_t}$ represents seasonality and ${h_t}$ represents holidays. Again, ${{ \epsilon }_t}$ is error term which takes in consideration any irregular changes that may not be accommodated by the model.

### LSTM

**Figure 1:**
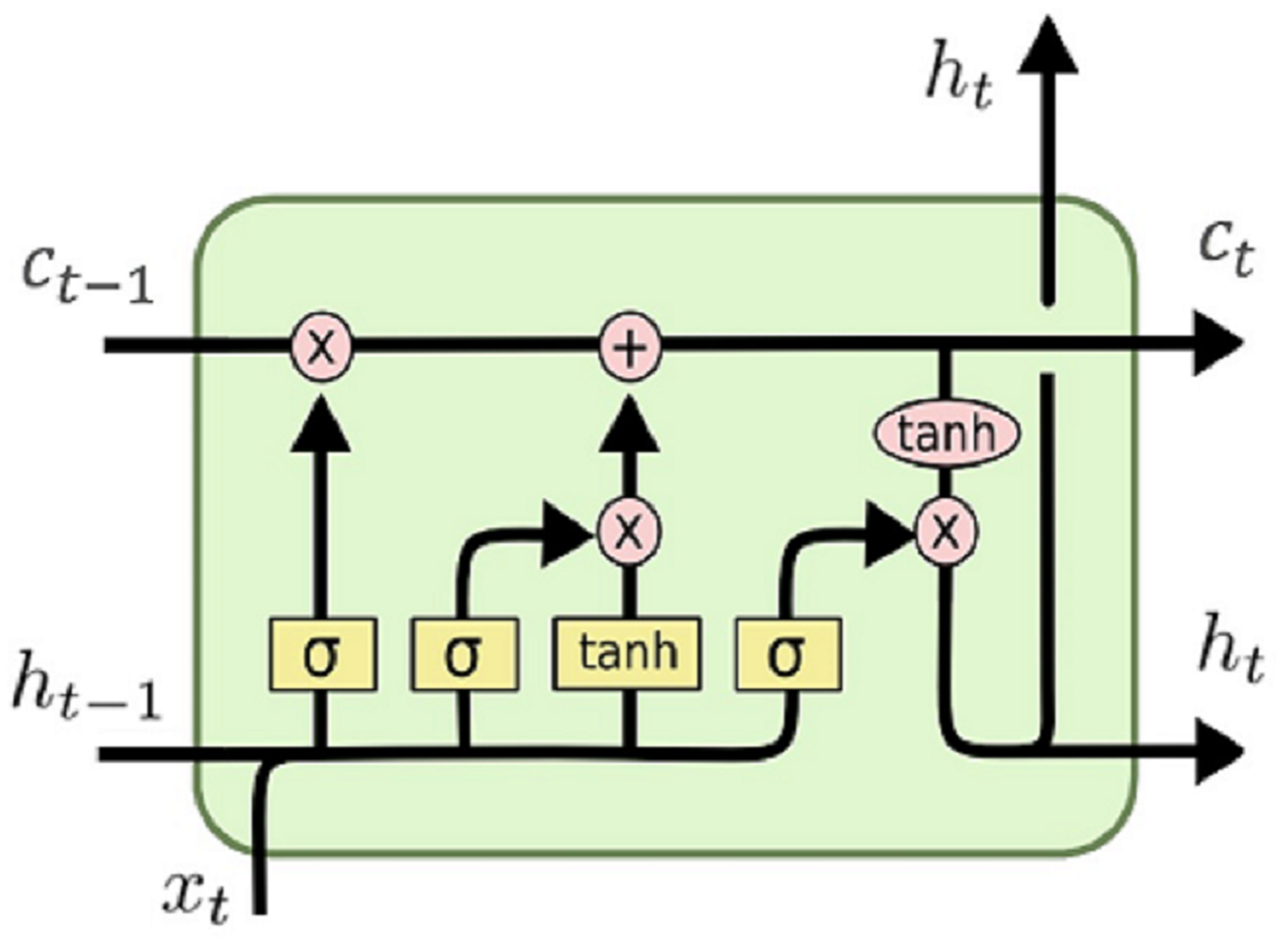
LSTM cell structure.

LSTMs are a special form of a recurrent neural network (RNN) and because of their memory structure, it is widely used in speech recognition, emotional analysis, text analysis, and time series forecasting. In LSTM model, the long sequence of data is remembered or stored by incorporating a gating mechanism. This gating mechanism uses some information from previous steps to produce output by evaluating a function. This output is used to modify the current LSTM cell state. There are three gate structures in LSTM cell; input gates, output gates, and forget gates, and the structure of an LSTM cell is shown in Fig. 1.

The forget gate is used to determine which information will be kept or not by using the following formula:

${f_t} = \sigma ({W_f}[{h_{t - 1}},{x_t}] + {b_f})$

where ${x_t}$ is input at time t, ${h_{t - 1}}$ is the output of previous cell, and $\sigma$ is sigmoid function. If the output of the forget gate is 1 (one), the information is kept in the cell state. After this step, sigmoid function creates a vector containing possible new values. Input gates decide which values will be updated and new candidate values vector $C_t^{\rm '}$ is created by following equation:

${i_t} = \sigma ({W_i}[{h_{t - 1}},{x_t}] + {b_i})$

$C_t^{\rm '} = tanh({W_c}[{h_{t - 1}},{x_t}] + {b_c})$

Now cell’s old state ${C_{t - 1}}$ is updated to new cell state ${C_t}$.

${C_t} = {f_t}*{C_{t - 1}} + {i_t}*C_t^{\rm '}$

Finally, we decide the output of the network and this output is based on our cell state. First, sigmoid layer is used to decide what parts of the cell state will be used and then, *tanh* function is used for cell state and multiplied by this sigmoid layer:

${o_t} = \sigma ({W_o}[{h_{t - 1}},{x_t}] + {b_o}).$

${h_t} = {o_t}*tanh({C_t}).$

**Figure 2:**
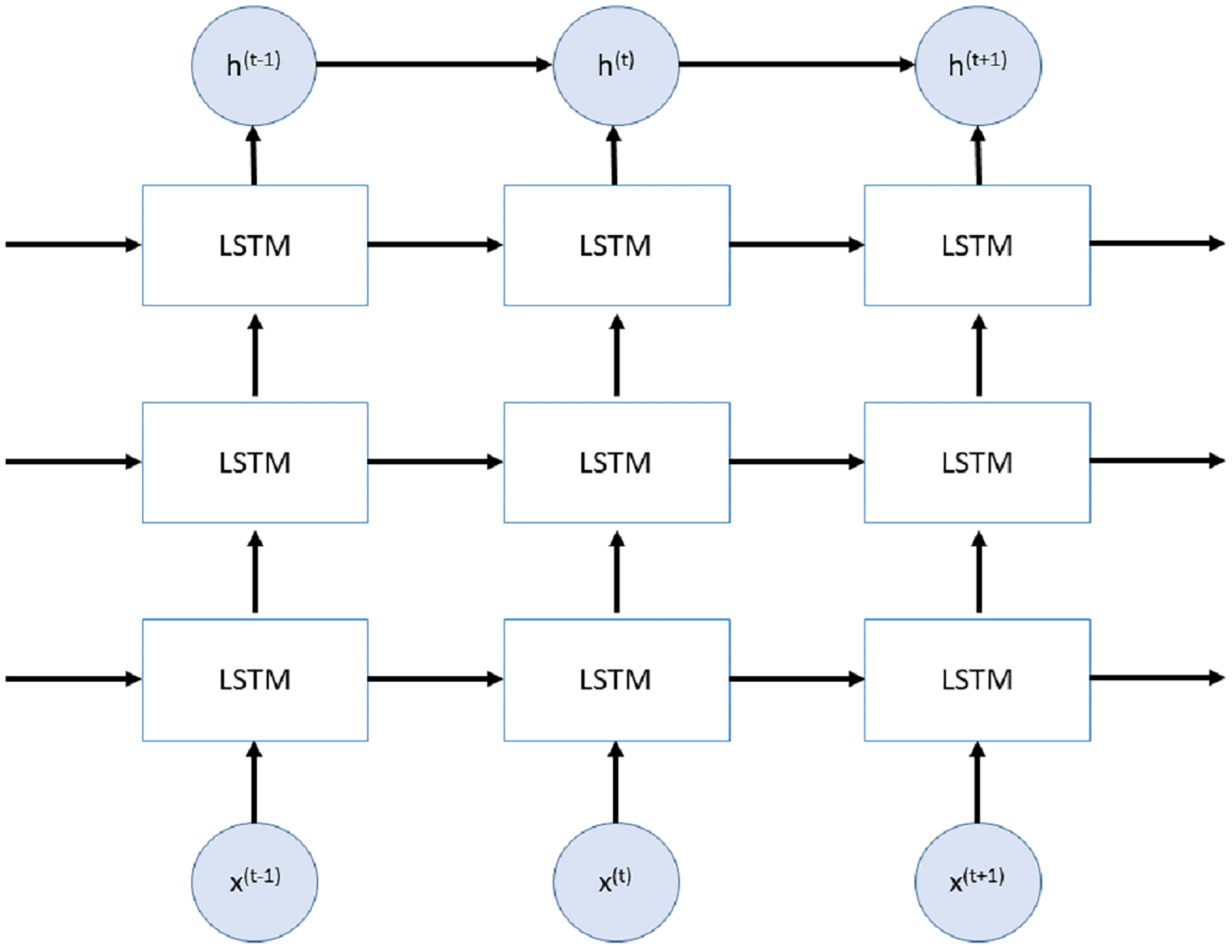
Stacked LSTM network.

Stacked LSTMs (SLSTMs) are presented in Graves, Mohamed & Hinton (2013) and use more than one LSTM layers. The first LSTM layer uses time series data as input and produces the output. This output is used to fed next LSTM layer. All LSTM layers have the same internal architecture with different number of units. An example of Stacked LSTM is shown in Fig. 2.

Traditional LSTMs uses only previous information in order to determine the next states. Bidirectional LSTMs (BDLSTMs) are developed to process information in both directions (Schuster & Paliwal, 1997). BDLSTMs are constructed by two separate hidden layers so that two independent LSTMs are used together to make bidirectional information passing possible at every time step. There are two different inputs of BDLSTM cell; input from previous steps and input from next step. By using two BDLSTM cell and combining their inputs-outputs, BDLSTM network can store information from both past and future.

## Proposed Hybrid Model

Time series data have linear and non-linear relationships. While statistical methods are eﬃcient at handling linear relationships in the time series, they cannot handle non-linear relationships (Martínez et al., 2018). On the other hand, neural network approaches can model linear and non-linear relationships but they depend on appropriate parameter selection and requires a long training time. However, the advantage of neural network(NN) models is that they can effectively handle non-linear relationships.

A hybrid model is implemented to cope with these weaknesses and work together for better performance. The proposed model tries to maintain the seasonality feature and also reduce the impact of this feature at the same time. In order to preserve seasonality, Prophet is adapted along with trend and noise. The Prophet model is mainly composed of three components: trend, seasonality, and holidays. In addition to this, the Prophet model has linear and non-linear growth trend functions to handle different types of time series. Hence, this helps the model fit data sets having different periodic change rules effectively.

**Figure 3:**
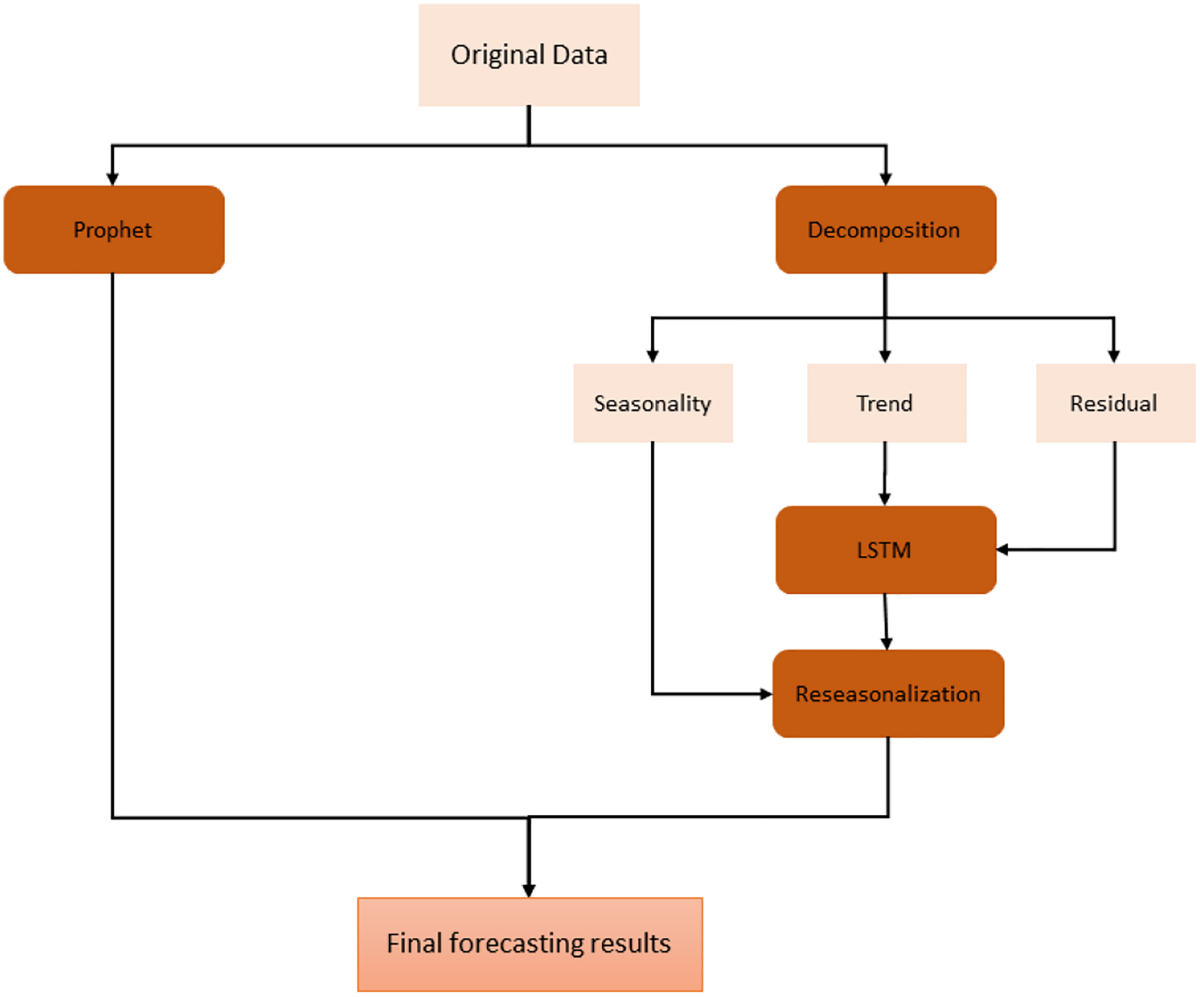
Proposed hybrid model.

On the other hand, we have used stacked bidirectional LSTM model which exploits the decomposition of time series data by removing the repeating temporal patterns. This decomposition and removal of the seasonality feature of time series data cause the system to handle irregular patterns in these data. For the decomposition, seasonal-trend decomposition based on the Loess (STL) method is applied to original time series data. The overall architecture of the proposed model is shown in Fig. 3.

First of all, original data is used to feed Prophet model. In order to use this model, the input data is first processed and modified since Prophet model expects only a formatted date and time series variable. After this preprocessing stage, some trend and seasonality parameters of the model are tuned to get fine results. The output of this model is simply forecasting values for the given specific time window.

**Figure 4:**
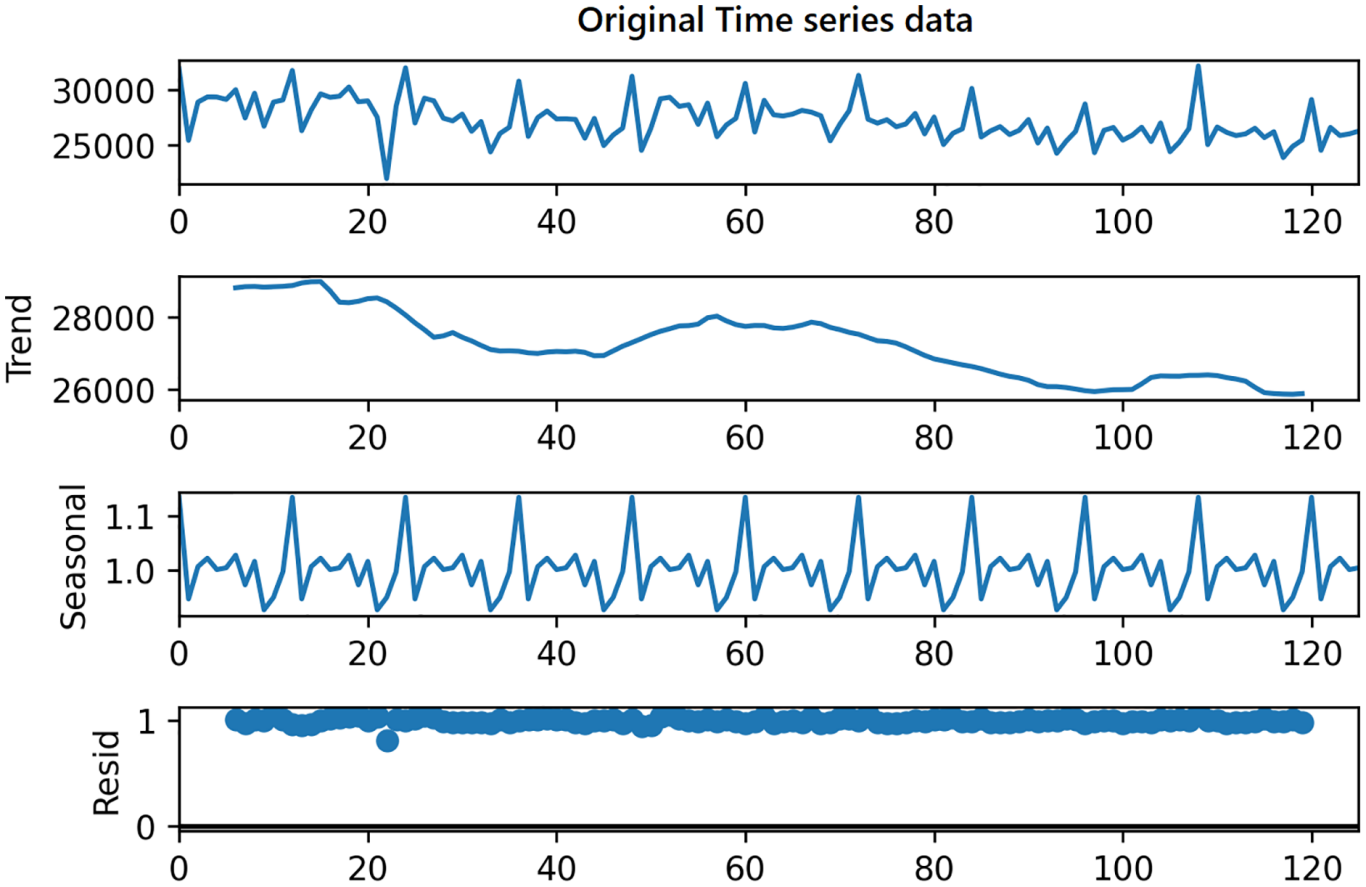
Decomposition of time series.

On the other hand, in order to use the same time series data as input for LSTM, seasonality information is extracted and removed from the original data. Time series data can exhibit a variety of patterns, and it is often helpful to split a time series into several components, each representing an underlying pattern category. An example of decomposition is demonstrated in Fig. 4. The figure shows the original signal and its components. The trend component refers to the general direction in which the time series is moving. Time series can have a positive or a negative trend, but can also have no trend. The seasonality component refers the repeating short-term cycle in the series. The residual or remainder component is what’s left of the time series data after removing its trend and seasonal components.

The decomposition algorithm simply extracts these three patterns from original data. The combination of the components in time series can be of two types; additive or multiplicative. In the additive time series, the components of the time series are added together to make the original time series. On the other hand, in the multiplicative time series, the components of the time series are multiplicative together.

In this study, in order to decompose the time series data, we have used *seasonal decompose* method of *statsmodels* Python library for additive decomposition shown in the following equation;

${Y_t} = {T_t} + {S_t} + {R_t}{\rm }$

where, ${T_t}$ represents trend, ${S_t}$ represents seasonality and ${R_t}$ represents residual. As a result, the original time series can be constructed by adding these patterns.

**Figure 5:**
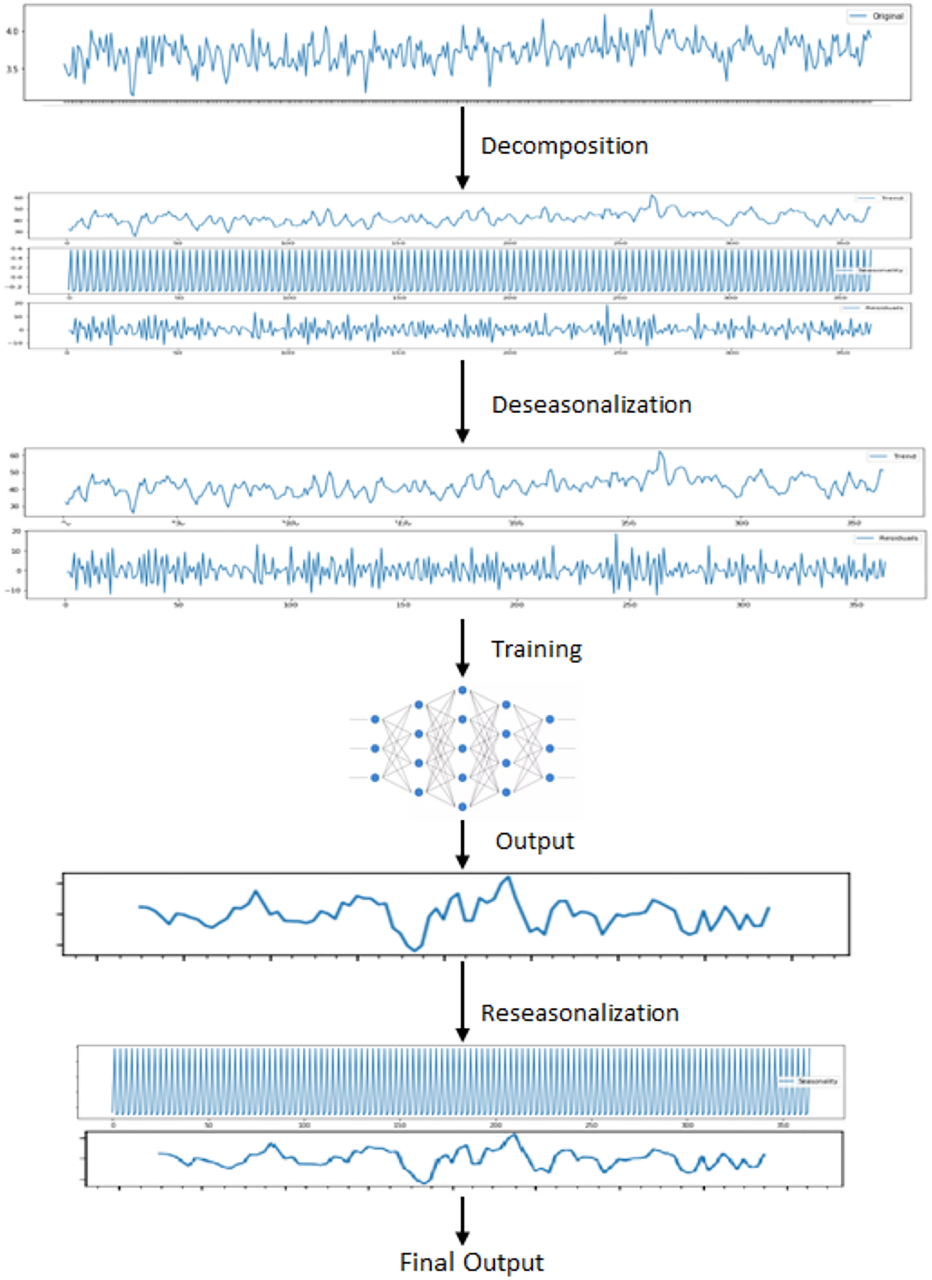
Steps for training LSTM.

After decomposition process, LSTM model is fitted to trend and residual components to train. Since this model ignores seasonal pattern, the reseasonalization process is applied after the prediction phase. In this process, the relevant seasonal components which are already extracted in decomposition stage are added to the forecasts generated by LSTM model. This is evaluated by a simple add function. The overall training phase is demonstrated in Fig. 5. In the last step, forecasts obtained from each model are aggregated using the combination of the equal weights in order to produce final forecasts.

### Model evaluation

The performance of a model can be tested by evaluating a comparison between the actual values and the predicted values. In this study, three performance metrics, namely the mean squared error (MSE), root mean square error (RMSE), mean absolute error (MAE) are used to evaluate the performance of each model. These metrics are calculated using the following equations;

$MSE = \displaystyle{1 \over n}\sum\limits_{i = 1}^n {{{(Y_{_i}^{\rm '} - {Y_i})}^2}}$

$RMSE = \sqrt {\displaystyle{{\sum\nolimits_{i = 1}^n {{{(Y_i^{\rm '} - {Y_i})}^2}} } \over n}} {\rm }$

$MAE = \displaystyle{1 \over n}\sum\limits_{i = 1}^n {|Y_i^{\rm '} - {Y_i}|}$

where $Y_i^{\rm '}$ is predicted value and ${Y_i}$ is actual value.

## Results

**Figure 6:**
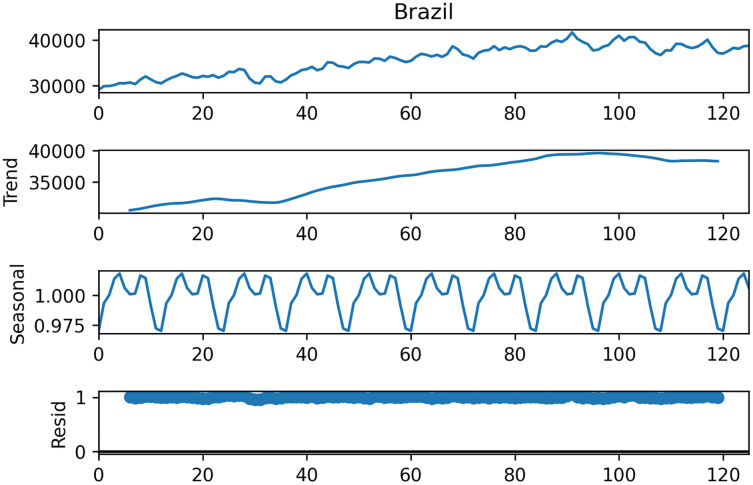
Decomposition of Brazilian data.

Within the scope of this study, the data set from the study de Oliveira & Cyrino Oliveira (2018) is used. This data set contains monthly data of total electric energy consumption (GWh) in seven different countries, namely Canada, France, Italy, Japan, Brazil, Mexico, and Turkey between 2006–2017 (126 months), and sample data is shown in Table 1. Figure 6 shows the STL decomposition results of the monthly electricity consumption in Brazil. The trend component of the electricity consumption of Brazil is increasing year by year. The time series data of other countries have similar trend and seasonality components.

**Table 1:** Sample data from energy consumption dataset of seven countries (GWh).

Date (mm/dd/yy)	Brazil	Canada	France	Italy	Japan	Mexico	Turkey
07/01/06	29,114	46,205	35,518	31,877	92,375	20,964	14,792
08/01/06	29,886	45,308	31,887	25,414	100,074	20,875	15,583
09/01/06	29,917	42,417	34,300	28,872	84,229	19,460	13,747
10/01/06	30,144	46,657	36,782	29,335	85,673	19,539	13,148
11/01/06	30,531	48,777	41,886	29,326	86,296	17,931	15,385
12/01/06	30,494	53,009	49,453	29,114	91,021	17,971	15,232

In order to show performance developments of our study, we compared our proposed hybrid system (Hybrid) with single stacked bidirectional LSTM model (BiLSTM) and also with deseasonalized LSTM model (deBiLSTM). Moreover, a Prophet model (Prophet) was used and compared with our hybrid model. In the training phase of the neural network part of the hybrid model, and also both BiLSTM and deBiLSTM models, the Adam optimizer was used. Mean squared error (MSE) was used as a loss function. We performed a series of tests for parameter selection since there is no global method to estimate these parameters. The selection of model parameters as completely data-dependent. Thus the parameters of the test with the best accuracy result are used throughout the experiments. Table 2 shows the topology for these models. Each model consisted of one input layer and three hidden layers for all countries. The Dropout layers were added to a model between these layers with 0.2 dropout rate. Dropout as used to reduce overfitting and improve the performance of the model Srivastava et al. (2014). At the end of the model, one dense layer as added in order to produce one-dimensional output, and batch size was set to 16 while the epoch parameter was set to 1,000 for training each model.

**Table 2:** Respective LSTM model topologies for each dataset.

Dataset	Method	Input	Hidden layer 1	Hidden layer 2	Hidden layer 3	Output	Sample length
Canada	Hybrid	(126,1)	16	8	4	(1)	14
	BiLSTM	(126,1)	16	8	4	(1)	14
	deBiLSTM	(126,1)	16	8	4	(1)	14
France	Hybrid	(126,1)	16	8	4	(1)	14
	BiLSTM	(126,1)	16	8	4	(1)	14
	deBiLSTM	(126,1)	16	8	4	(1)	14
Japan	Hybrid	(126,1)	32	16	8	(1)	5
	BiLSTM	(126,1)	32	16	8	(1)	5
	deBiLSTM	(126,1)	32	16	8	(1)	5
Mexico	Hybrid	(126,1)	64	32	16	(1)	24
	BiLSTM	(126,1)	64	32	16	(1)	24
	deBiLSTM	(126,1)	64	32	16	(1)	24
Brazil	Hybrid	(126,1)	8	8	4	(1)	5
	BiLSTM	(126,1)	8	8	4	(1)	5
	deBiLSTM	(126,1)	8	8	4	(1)	5
Turkey	Hybrid	(126,1)	16	8	4	(1)	14
	BiLSTM	(126,1)	16	8	4	(1)	14
	deBiLSTM	(126,1)	16	8	4	(1)	14
Italy	Hybrid	(126,1)	32	16	8	(1)	5
	BiLSTM	(126,1)	32	16	8	(1)	5
	deBiLSTM	(126,1)	32	16	8	(1)	5

To be able to use time series data with these LSTM models, we should transform these data into a structure of samples with input and output components. The Keras deep learning library provides the TimeseriesGenerator to automatically transform time series data into samples. TimeseriesGenerator uses the length parameter in order to define the sample length used to train the model. This parameter as used for predicting the next value after sample input which has (length) number of elements. For both models, different sample lengths which give the best accuracy results were used and these values are shown in the ‘Sample Length’ column of Table 2. Table 3 shows the performance results of models for each country.

**Table 3:** Performance results of models for each country.

Dataset	Method	MAE	MSE	RMSE
Canada	Hybrid	1,778.090	5,514,174.558	**2,348.227**
	Prophet	2,692.760	16,970,198.845	4,119.490
	BiLSTM	2,024.409	7,131,390.874	2,670.466
	deBiLSTM	4,939.208	33,110,916.113	5,754.208
France	Hybrid	1,536.671	4,611,178.316	**2,147.365**
	Prophet	3,012.561	15,460,298.534	3,931.958
	BiLSTM	1,715.494	4,736,708.254	2,176.398
	deBiLSTM	6,313.654	47,940,856.736	6,923.933
Japan	Hybrid	2,802.822	11,420,135.400	**3,379.369**
	Prophet	4,921.811	31,072,199.315	5,574.244
	BiLSTM	7,470.529	84,741,633.077	9,205.521
	deBiLSTM	5,862.992	47,186,452.464	6,869.239
Mexico	Hybrid	8,80.816	1,105,985.936	**1,051.658**
	Prophet	1,029.039	1,589,788.345	1,260.868
	BiLSTM	881.306	1,224,851.574	1,106.730
	deBiLSTM	2,186.271	6,005,540.182	2,450.620
Brazil	Hybrid	1,519.002	2,642,345.014	1,625.529
	Prophet	2,733.089	8,786,595.659	2,964.219
	BiLSTM	903.928	1,200,980.218	1,095.892
	deBiLSTM	803.921	1,000,117.467	**1,000.058**
Turkey	Hybrid	1,122.314	1,844,760.862	1,358.219
	Prophet	706.916	916,967.034	**957.583**
	BiLSTM	1,792.026	5,655,776.258	2,378.187
	deBiLSTM	2,177.616	7,583,899.725	2,753.888
Italy	Hybrid	845.159	1,225,318.038	**1,106.940**
	Prophet	1,520.558	3,612,153.530	1,900.566
	BiLSTM	1,965.405	4,830,733.821	2,197.893
	deBiLSTM	989.882	2,667,179.095	1,633.150

Note: Bold text indicates best results.

**Figure 7:**
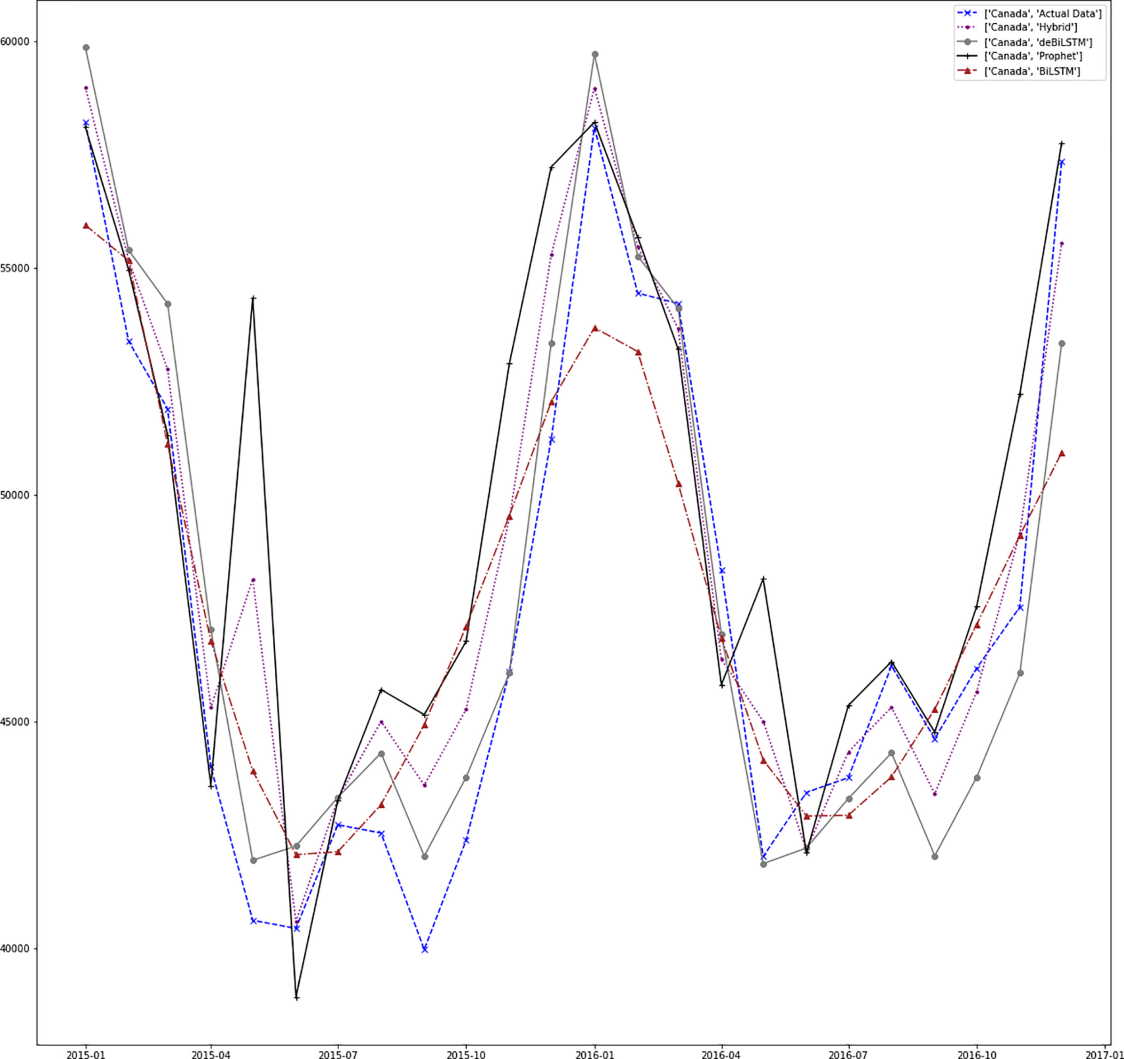
Prediction results for Canada.

**Figure 8:**
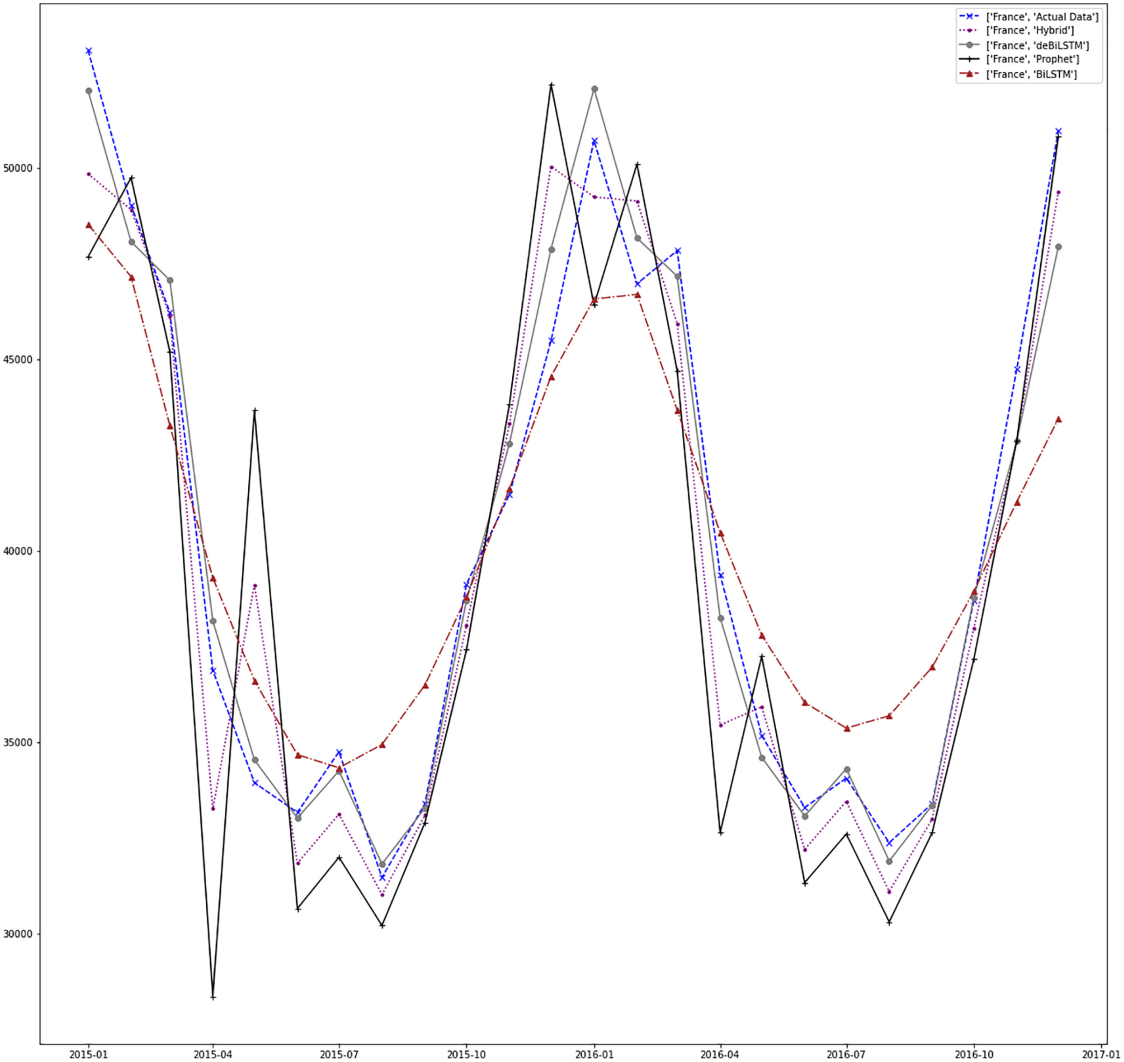
Prediction results for France.

**Figure 9:**
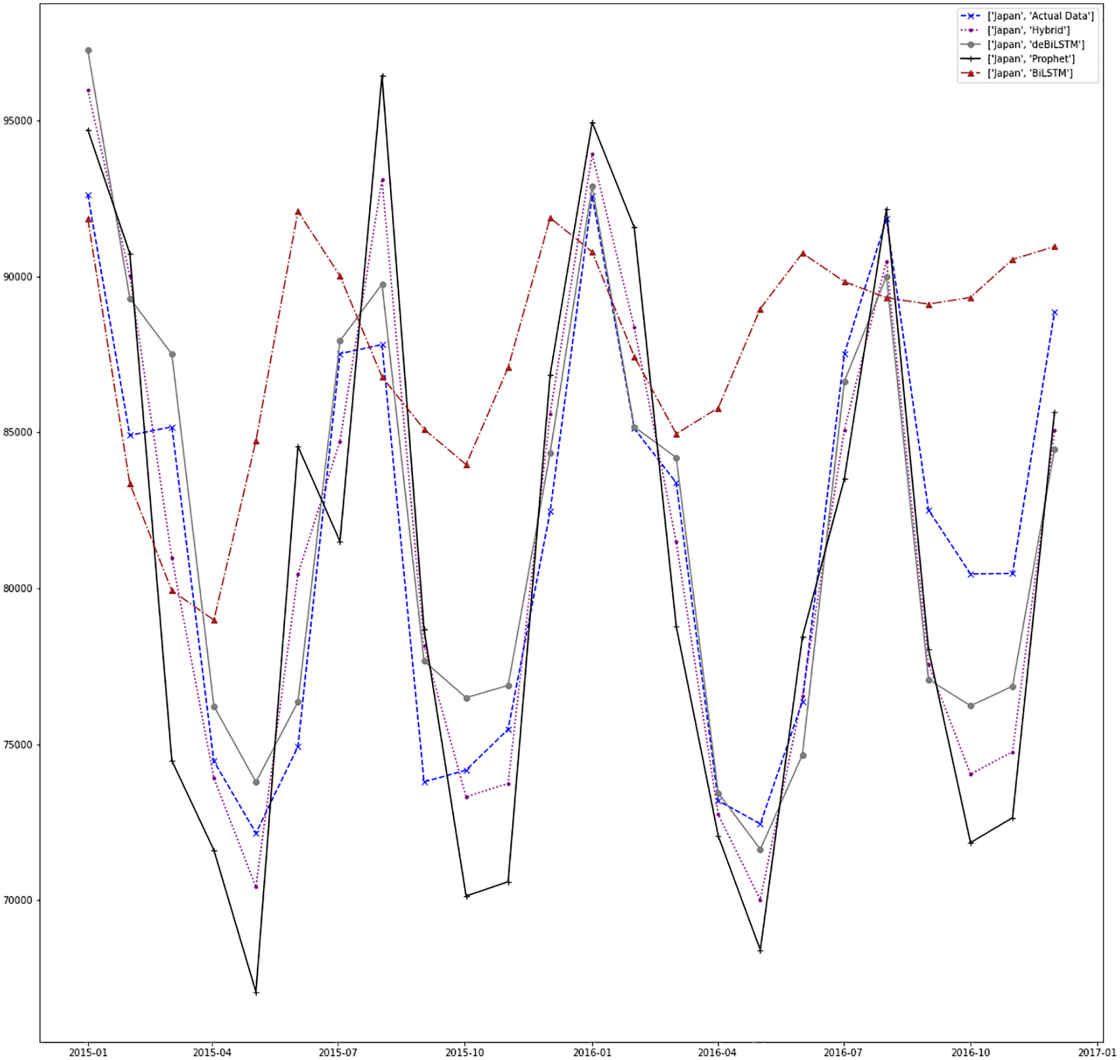
Prediction results for Japan.

**Figure 10:**
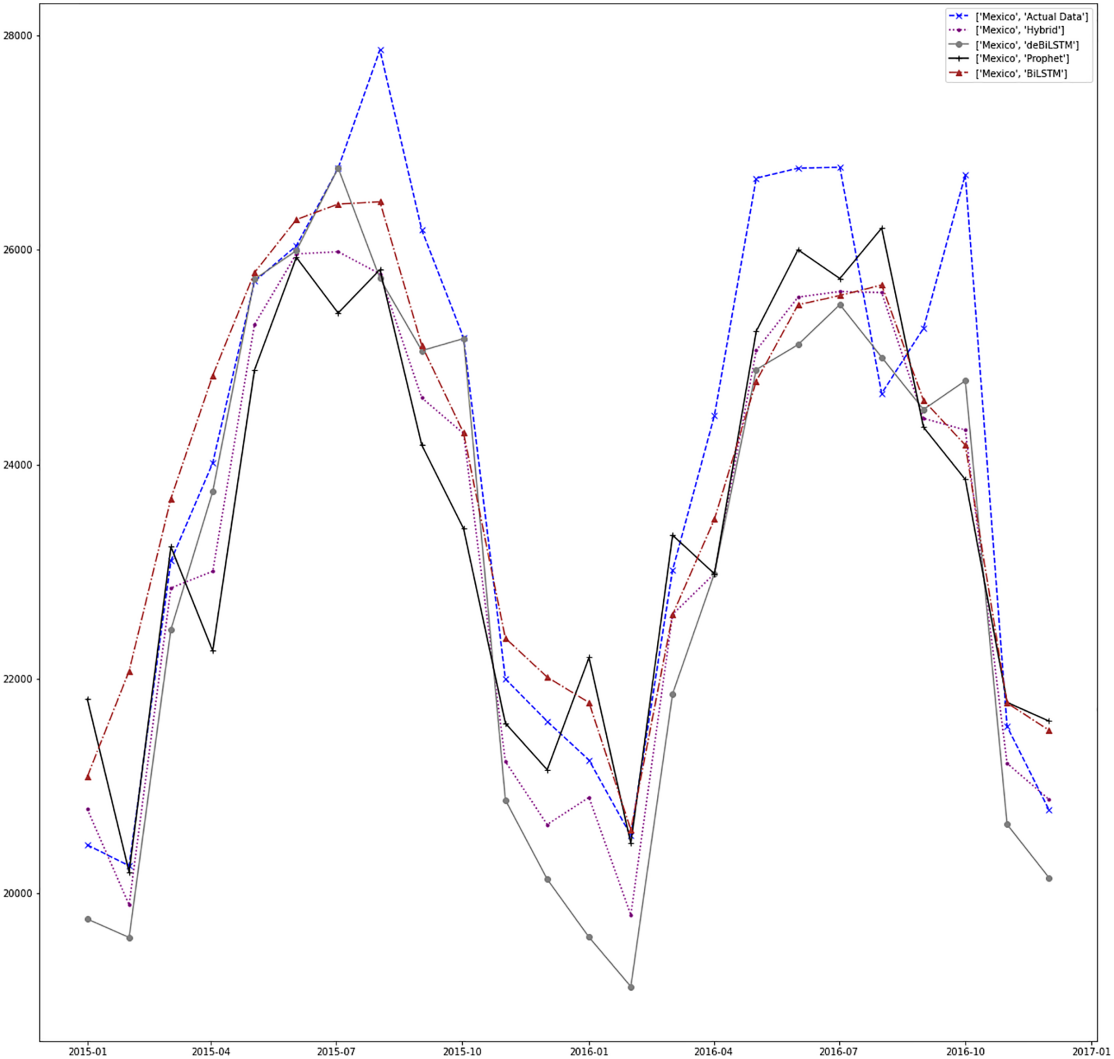
Prediction results for Mexico.

**Figure 11:**
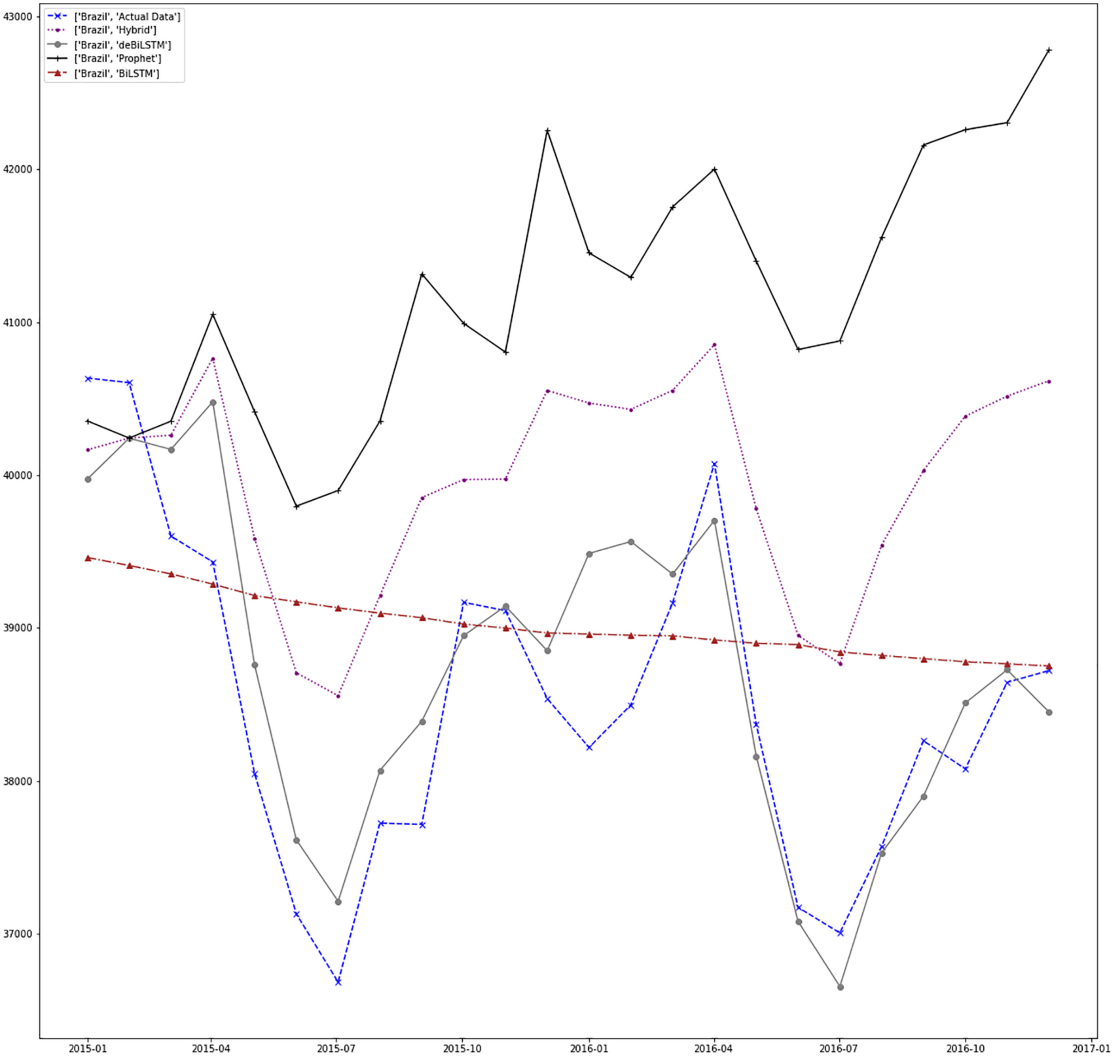
Prediction results for Brazil.

**Figure 12:**
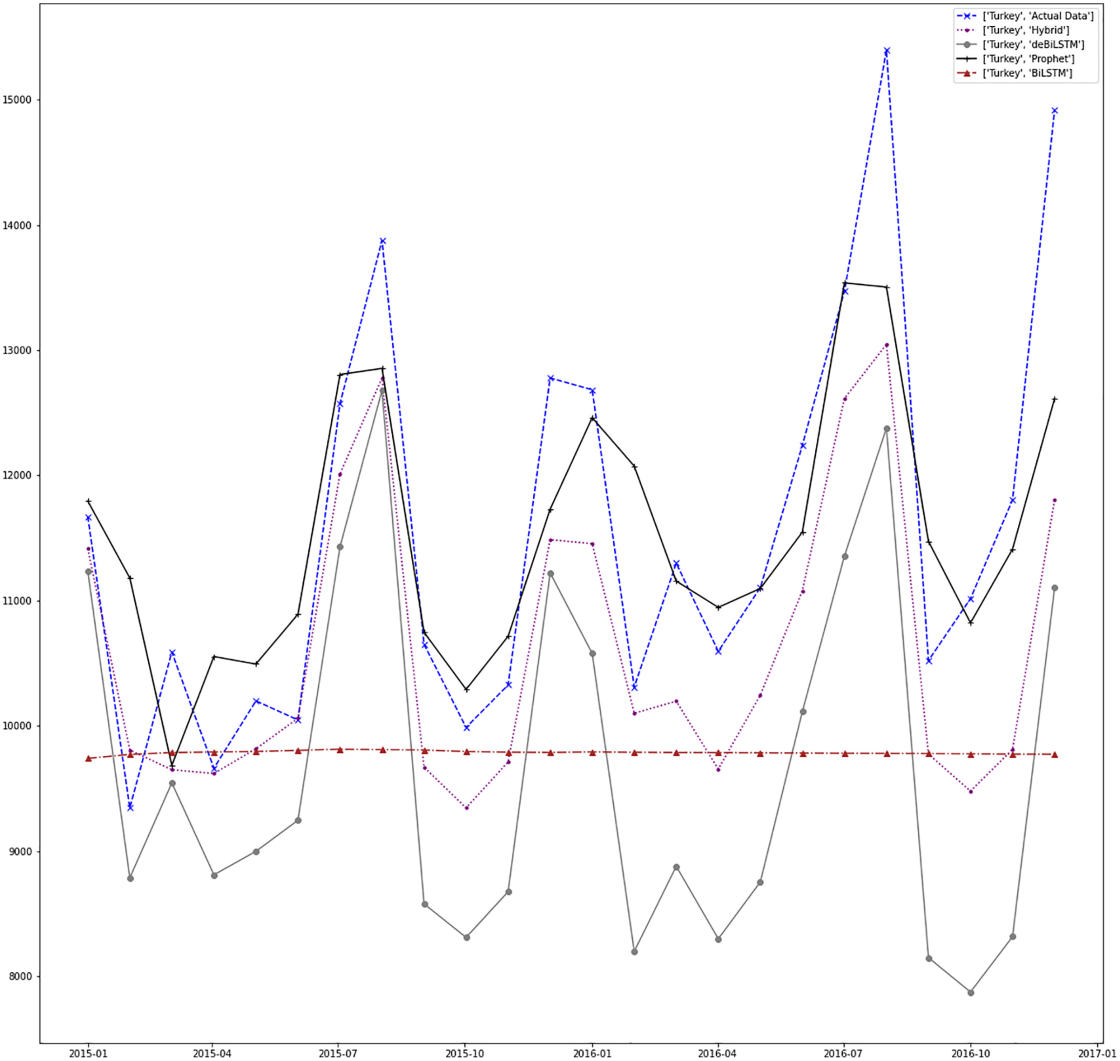
Prediction results for Turkey.

**Figure 13:**
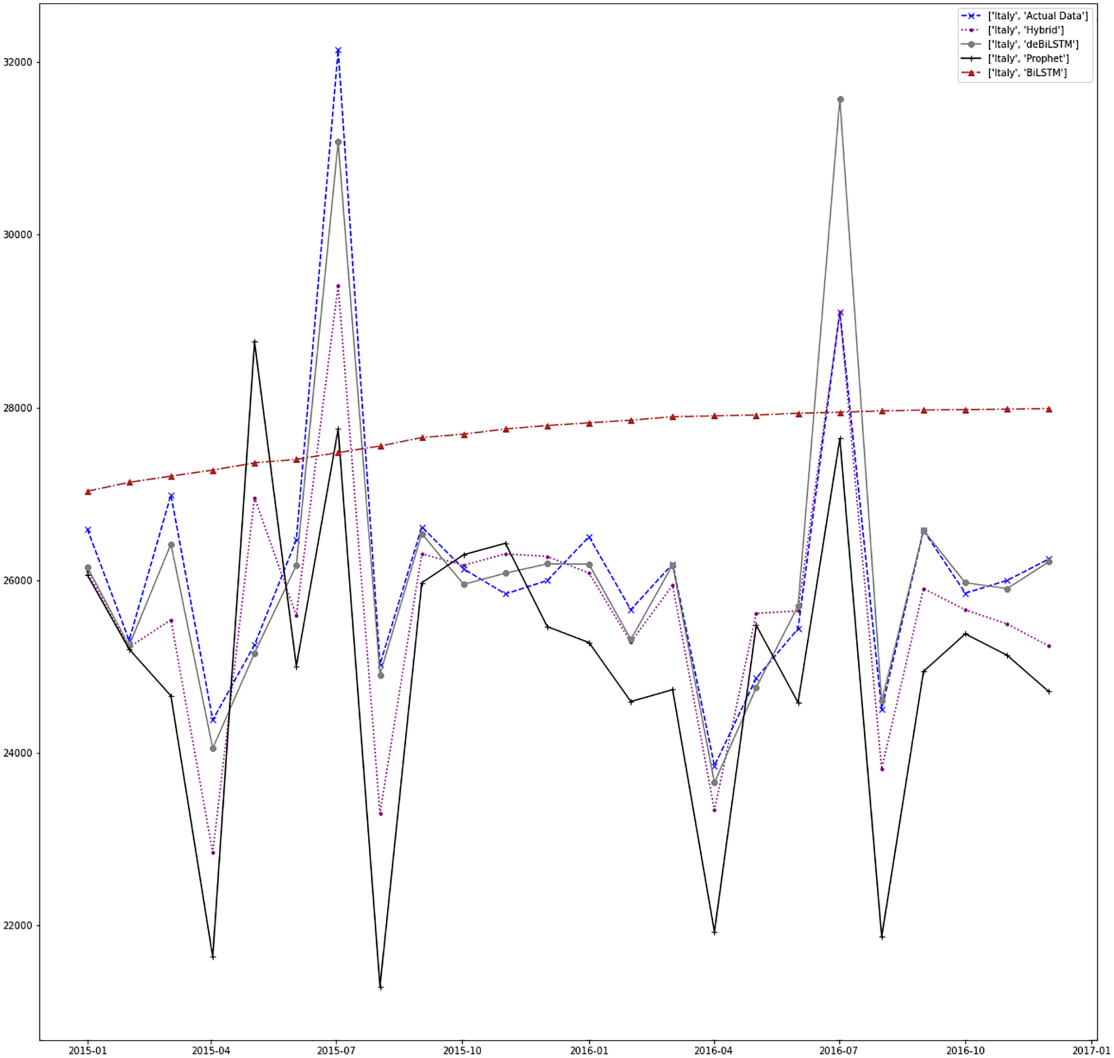
Prediction results for Italy.

Figures 7–13 demonstrate the forecasts generated by each model and also actual data. From the figures and results, we can conclude that using the proposed hybrid model with a deseasonalization approach can substantially improve the forecast accuracy for most of the data sets. The only exceptions are for Turkey, using only Prophet model has better performance results, while for Brazil, the best forecast values are achieved using neural networks only. For the other five countries, the proposed hybrid model performed better.

We also compared the hybrid model with the state-of-art techniques. ARIMA is a state-of-art statistical technique that is widely used in time series domain. Support vector regression (SVR) uses the basic idea of support vector machines for regression problems and it is widely used for learning from training data in order to generate decision boundaries in non-linear space (Smola & Schölkopf, 2004). The Holt-Winters additive model (Winters, 1976) is the most commonly used method since it is simple, effective, and has computing eﬃciency. It tries to capture seasonality patterns from time series data.

Empirical Mode Decomposition (EMD) is a signal processing method used for the decomposition of signals (Huang et al., 1998). It is a very eﬃcient technique for dealing with non-linear data (Lahmiri, 2015). Recently, EMD is adapted to RNNs for fore-casting of time series. In Bedi & Toshniwal (2018), EMD-LSTM is applied to energy consumption data. EMD-GRU is adapted in Gao et al. (2019) for short-term electricity load forecasting. Table 4 shows this comparison results in terms of RMSE metric. Our method performs better than other methods for Canada, Brazil, and Japan. Despite standard ARIMA and EMD-LSTM models performing better than our model for the other countries, our model results are very close to these results.

**Table 4:** Comparison with other models.

Dataset	Method	RMSE
Canada	Hybrid	**2,348.227**
	ARIMA	2,718.240
	SVR	4,977.226
	Holt-Winters	2,474.736
	EMD-LSTM	2,514.770
	EMD-GRU	2,912.871
France	Hybrid	**2,147.365**
	ARIMA	2,865.827
	SVR	3,881.359
	Holt-Winters	2,463.391
	EMD-LSTM	2,571.966
	EMD-GRU	2,748.025
Japan	Hybrid	**3,179.369**
	ARIMA	3,267.591
	SVR	6,883.180
	Holt-Winters	3,345.802
	EMD-LSTM	3,401.124
	EMD-GRU	3,299.705
Mexico	Hybrid	1,051.658
	ARIMA	968.572
	SVR	4,538.936
	Holt-Winters	1,558.574
	EMD-LSTM	**801.477**
	EMD-GRU	1,022.981
Brazil	Hybrid	1,625.529
	ARIMA	1,943.011
	SVR	2,160.605
	Holt-Winters	2,961.887
	EMD-LSTM	**907.844**
	EMD-GRU	1,871.742
Turkey	Hybrid	1,358.219
	ARIMA	**681.329**
	SVR	1,887.737
	Holt-Winters	707.484
	EMD-LSTM	1,501.665
	EMD-GRU	1,444.853
Italy	Hybrid	1,106.940
	ARIMA	1,221.386
	SVR	1,613.364
	Holt-Winters	803.252
	EMD-LSTM	**768.841**
	EMD-GRU	998.125

Note: Bold text indicates the best results.

## Conclusion

In this study, we proposed a hybrid forecasting model for time series data to improve prediction accuracy. Our model is based on two different sub-models; a Prophet model for preserving seasonality information of time series and a neural network model (stacked bidirectional LSTM) for a deseasonalized version of this data. Thus, our model is capable of handling seasonality patterns and at the same time, it tries to reduce the impact of this seasonality in the prediction phase. By decomposition of time series data into its patterns, seasonality patterns can be extracted and removed from the original data and deseasonalized data is used in the training phase of the neural network model to have less training time. The extracted seasonality information is added back to the prediction output of the neural network model and final prediction results are produced by merging prediction results generated by both sub-models.

We have evaluated the proposed model with real-world data set, containing monthly energy consumption values of seven countries for ten and half years. The model is compared with the single Prophet model, single stacked bidirectional LSTM model for original data, and also single stacked bidirectional LSTM model for deseasonalized data. The study showed that the decomposition of time series influences the final prediction accuracy because of the removal of irregular fluctuations. In addition to this, the results indicate that using a hybrid model has better prediction results than single models. Moreover, our model is compared with state-of-art techniques including ARIMA, support vector regression (SVR), Holt-Winters exponential smoothing method, EMD-LSTM, and EMD-GRU for time series data. The accuracy results show that our method outperforms these methods for some countries in our dataset while for other countries, it has competitive performance.

In the future, we will try to use different data sources, with seasonality patterns. In addition, various types of RNNs will be adapted to explore the generalization and optimization of the hybrid mechanism.

## Supplemental Information

10.7717/peerj-cs.1001/supp-1 Supplemental Information 1 Code developed on Jupyter notebook for model implementation and dataset.

## Additional Information and Declarations

### Data Availability

The following information was supplied regarding data availability: The implementation of model and energy data set are available in the Supplemental File.
